# “Selected Papers from the 2nd Ellisras Longitudinal Study and Other Non-Communicable Diseases Studies International Conference” Special Issue Editorial

**DOI:** 10.3390/children8020146

**Published:** 2021-02-16

**Authors:** Kotsedi Daniel Monyeki

**Affiliations:** Department of Physiology and Environmental Health, University of Limpopo, Private Bag X1106, Sovenga 0727, South Africa; kotsedi.monyeki@ul.ac.za

**Keywords:** children, education, non communicable diseases, physical education, health promotion

## Abstract

Epidemics of non-communicable diseases (NCDs) are presently emerging and on the increase in South Africa. It is increasingly recognized that the occurrence of adult chronic disease are influenced by factors operating from childhood, which are sustained throughout the individual’s life course. Increased risk may start in infancy or even before birth and will continue to be influenced by health related behavior during adulthood. The academic level of people in the community influence the level of their health status. Commitment to the promotion of health through prevention, education, and suitable management is the building block for creating a healthy society. The community must make strides to shift from traditional knowledge and medication, and seek new innovative ways of addressing issues facing the population with regard to obesity, overweight, hypertension health, smoking cessation, alcohol abuse, and low physical activity in line with a healthy living lifestyle. The NCDs pose health problems in South Africa and deserve more attention. Poor control of obesity, hypertension, and diabetes, to name just a few, only adds to the current problems. The South African government and the business sector of South Africa should provide safe walking/riding trails in the cities and in rural area to combat emerging NCDs that are killing our community members indiscriminately without considering race, gender, age, and place of residence. Compulsory introduction of physical education lessons to all public schools cannot be over emphasized in the current escalating NCD situation in South Africa.

## 1. Introduction

The Special Edition of selected papers from the 2nd Ellisras Longitudinal Study and Other Non-Communicable Diseases Studies International Conference (ELSONCDIC) held in the University of Limpopo and Letlamoreng Village, Lephalale, South Africa during the period 3–5 December 2019 were published in Children in 2020 (https://www.mdpi.com/journal/children/special_issues/Ellisras_ncd_study). Five outstanding articles from a plethora of esteemed scientists, scholars, and postgraduate students who were willing to share their wealth of knowledge with their peers and ordinary members of the Ellisras/Lephalale community were collated.

This 2nd ELSONCDIC provided a unique inclusive platform for discussion by ordinary members of the Ellisras/Lephalale community, expert scholars, postgraduate students, and experienced professionals from all over the world, offering a truly special international networking experience. A comprehensive and interactive program in which participants cultivated their cross-cultural and communication skills while highlighting different topics related to poverty and cardiovascular diseases was achieved. In addition, the South African National Development Plan (SANDP) version of 2030 [1] declares that full participation of the community in changing their lifestyle is the key, given that societal risk conditions are more important than that of the individual. As the guest editor, may I thank the authors for the quality of the papers, which shows the best indicator of the intellectual vigor and pedagogical excellence of their work.

## 2. Perspective: 2nd Ellisras Longitudinal Study and Other Non-Communicable Diseases Studies International Conference

### 2.1. Study Design and Hierarchy of Evidence Based Studies for Public Health

**Figure 1:**
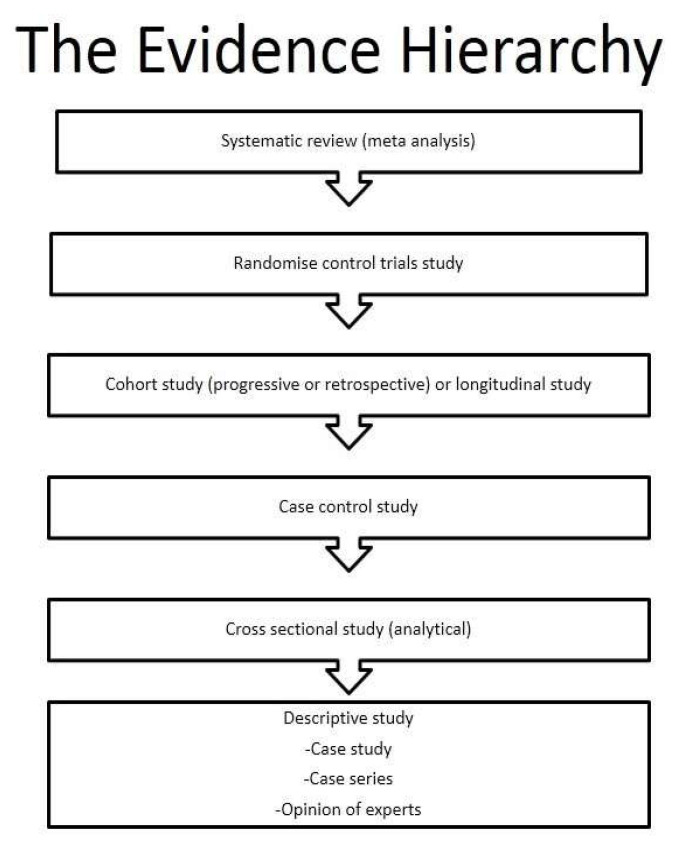
Studies showing the hierarchy of research evidence-based health care [6,7].

The NCD profile has been suggested to be changing rapidly amongst the rural South African population. However, evidence to sustain these changes is still very limited. Globally, Africa is expected to experience the highest increase in NCD related mortality, with about 46% of all expected mortality attributed to NCDs by 2030 [2,3]. Exposure to known risk factors account for about two-thirds of premature NCD deaths with an estimated half of NCD deaths attributed to weak health systems and poverty in sub Saharan Africa [4]. Well formulated cohort studies in Africa like the Ellisras Longitudinal Study (ELS) as recently published by Makgae et al. [5] could answer major questions relating to the changing magnitude of the NCD risk factor profile in Africa from childhood to adulthood over time. The ELS aimed to track the role of lifestyle and biological risk factors in determining adverse health outcomes, in particular, the development of NCDs, obesity, hypertension and diabetes in a cohort of rural South African children (born between 1986 to 1993) followed over time to date. At baseline (November 1996), 2225 children were selected for the study, using cluster random sampling techniques [5]. Such studies are amongst the top studies that provide reliable and valid information as outlined in the hierarchy of research evidence based on the health care chart as seen in Figure 1 [6,7]. My thanks go to the Amsterdam Growth and Health Longitudinal study team members for their input and support for the existence of the ELS. More such studies are encouraged in Africa given the few studies that currently exist in rural areas of South Africa.

The SANDP vision for 2030 [1] highlights key recommendations for reducing the prevalence of NCDs by 28% in 2030, which are all mirrored in the World Heart Federation, though they focus on reducing the NCD prevalence by 25% in 2025 [8]. The targeted diseases include among others, cardiovascular diseases, diabetes, cancer, and chronic respiratory diseases. The risk factors to be targeted include tobacco smoking, physical inactivity, raised blood pressure, raised blood glucose, obesity, and raised cholesterol. The ELS and results of other studies of NCDs presented at the 2nd ELSONCDIC provided a unique opportunity of mapping some of these changes in vulnerable adolescent and young adults in South Africa.

### 2.2. Growth Patterns of Rural South African Children

Physical growth in South African children has been the subject of several studies during the past years and these have mostly been cross-sectional [9,10,11]. These studies revealed that growth rates of height and weight of children are considered to be the best dynamic indicators of nutritional and health status. Little longitudinal data of growth on the same group of children during childhood, adolescence, and adulthood have been reported from undernourished populations of South Africa in that period. Recently, Nembidzane et al. [12] reported that age at peak height velocity for Ellisras rural children was 14.45 years for boys and 11.82 years for girls; the age that Ellisras rural girls had their peak height velocity was way earlier than Ellisras rural boys did by an estimated 2.63 years. These ELS results were comparable with other results, particularly those of the Edinburgh children [13]. However, earlier puberty is more likely a result of adiposity gain in childhood than a cause of adiposity gain in adulthood in females [14] as was the case amongst Ellisras girls. This was evident in the high prevalence of obesity and hypertension amongst girls compared to boys as they grew older [15,16]. Low 25-hydroxyvitamin D was correlated with waist girth, and therefore with adiposity [17].

Knowledge of the development of NCDs over time is therefore important, given the long incubation period of the NCDs. Educational programs should be implemented to educate the communities and the nation at large about the dangers posed by the NCDs over time.

### 2.3. Health Status of Rural South African Children

The development of obesity/overweight has been linked to four critical stages or sensitive periods: intrauterine life, infancy, the periods of adipose rebound (5–7 years), and adolescence [18]. The onset of obesity or overweight during one or more of these periods appears to increase the risk of overweight related conditions such as hypertension later in life [19].

Sebati et al. [20] and Pisa et al. [21] reported high % body fat in girls compared to boys and a significant association between systolic blood pressure and waist girth even after adjusting for age and gender amongst urban South African children. Furthermore, Matjuda et al. [22] reported that obesity and hypertension were associated with renal-cardiovascular disease risk, while oxidative stress showed a possible association with obesity in six to nine year old South African children of African descent. In the ELS results, binge drinking was reported to be significantly associated with low levels of high-density lipoprotein cholesterol (HDL-C) (OR = 2.64, 95% CI = 1.23–5.65), after being adjusted for smoking, age, and gender [23], while the prevalence of tobacco product use in the ELS was reported to increase with increasing age [24].

This suggests that South African children, in the midst of the poverty they live under, may be becoming more prone to developing NCDs, and therefore may require early intervention for the prevention of NCDs in the near future given the high cost of medicine today in the shrinking South African economy due to COVID-19.

### 2.4. Dietary Patterns and Nutritional Status of Rural South African Children

Malnutrition (height-for-age, weight-for-age, and Body Mass Index (BMI)-for-age) z scores have been used in many studies of the rural South African populations. The prevalence of stunting, thinness, and wasting was reported to be high amongst rural South African children [25,26,27], with no signs of declining soon.

Recently, Modjadji et al. [27] reported that consumption of a diversified diet was associated with lower odds of being stunted [AOR = 0.25, 95%CI: 0.10 to 0.92] among four-year olds, while in the unadjusted model, 5-year-olds had lower odds of being underweight [OR = 0.32, 95%CI: 0.57 to 0.07] in the North West Province of South Africa. Through previous studies, it is a known fact that rural South African children enjoy two meals a day of low energy intake [28]. However, the risk of cardiovascular disease increases as these children from rural areas eat too much of high fat diet over time during their sporadic occasions (weddings, funerals, etc.) in the area [21,29]. It is a known fact that fat cells never really disappear as they decrease or shrink when one loses weight [30,31].

This poses a threat later in life, which requires a significant change in the lifestyle of rural South African children at an early age. Healthy lifestyles should be advocated to all community members once such a platform presents itself.

## 3. Conclusions

More quality study design should be made available to provide evidence in combating NCDs in the South African population. Many changes are taking place in South Africa today including the adoption of western diets, excessive use of contemporary tobacco products, and an increasing level of alcohol usage by children, which is of real concern. Changing lifestyles away from unhealthy risk behaviors should be encouraged to individuals, which will ultimately affect community members.

To succeed in changing the lifestyle of an individual first requires the eradication of illiteracy by health professionals, academics, and scholars in terms of providing primary health information to these sectors of the community. The community must make strides to shift from traditional knowledge and medication, and seek new innovative ways of addressing issues facing the population with regard to obesity, overweight, hypertension, health, smoking cessation, alcohol abuse, and low physical activity in line with a healthy living lifestyle.

The South African government and the business sector of South Africa should provide safe walking/riding trails in the cities and in rural areas to combat emerging NCDs that are killing our community members indiscriminately without considering race, gender, age, and place of residence. Introduction of compulsory physical education lessons in all public schools cannot be over emphasized.

## Data Availability

Not applicable.
